# Fluorescent Silicate Materials for the Detection of Paraoxon

**DOI:** 10.3390/s100302315

**Published:** 2010-03-19

**Authors:** Brandy J. Johnson, Brian J. Melde, Cassandra Thomas, Anthony P. Malanoski, Iwona A. Leska, Paul T. Charles, Damon A. Parrish, Jeffrey R. Deschamps

**Affiliations:** 1 Center for Bio/Molecular Science and Engineering, Naval Research Laboratory, Washington, DC 20375, USA; E-Mails: brian.melde@nrl.navy.mil (B.J.M.); cassandrathomas06@yahoo.com (C.T.); anthony.malanoski@nrl.navy.mil (A.P.M.); paul.charles@nrl.navy.mil (P.T.C.); damon.parrish@nrl.navy.mil (D.A.P.); jeff.deschamps@nrl.navy.mil (J.R.D.); 2 NOVA Research Incorporated, Alexandria, VA 22308, USA; E-Mail: iwona.leska.ctr@nrl.navy.mil

**Keywords:** mesoporous, organosilica, fluorescence, porphyrin, hierarchical, detection, organophosphate

## Abstract

Porphyrins are a family of highly conjugated molecules that strongly absorb visible light and fluoresce intensely. These molecules are sensitive to changes in their immediate environment and have been widely described for optical detection applications. Surfactant-templated organosilicate materials have been described for the semi-selective adsorption of small molecule contaminants. These structures offer high surface areas and large pore volumes within an organized framework. The organic bridging groups in the materials can be altered to provide varied binding characteristics. This effort seeks to utilize the tunable binding selectivity, high surface area, and low materials density of these highly ordered pore networks and to combine them with the unique spectrophotometric properties of porphyrins. In the porphyrin-embedded materials (PEMs), the organosilicate scaffold stabilizes the porphyrin and facilitates optimal orientation of porphyrin and target. The materials can be stored under ambient conditions and offer exceptional shelf-life. Here, we report on the design of PEMs with specificity for organophosphates and compounds of similar structure.

## 1. Introduction

**Figure 1.**
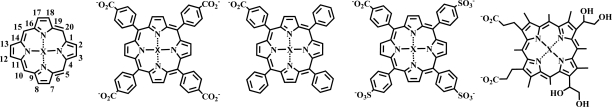
Structures of the porphyrin parent compound and those porphyrins used in the presented studies; from left to right: porphine, meso-tetra(4-carboxyphenyl) porphine (C_4_TPP); 5-mono(4-carboxyphenyl)-10, 15, 20-triphenyl porphine (C_1_TPP); meso-tri(4-sulfonatophenyl)mono(4-carboxyphenyl) porphine (C_1_S_3_TPP); and Deuteroporphyrin IX 2,4 bis-ethylene glycol (DIXEG).

Fluorescence-based detection of low molecular weight compounds can be accomplished using indicators of widely varying selectivity. Antibody-based recognition, for example, may offer highly selective binding of a single analyte. Other approaches may use a “fingerprint” response pattern across a number of indicators of lower selectivity. The effort described here employs a porphyrin for detection of targets. Porphyrins can be designed to offer semi-selective binding characteristics through altering the peripheral substituent groups of the compounds or through incorporation of a metal via coordination to the central nitrogen atoms. They have been employed for detection of a number of analytes from oxygen [1] to DNA [2]. Here, we do not seek to offer a review of these numerous efforts, instead we refer the reader to reviews of the topic [3,4]. The unique spectrophotometric characteristics of porphyrins result from their highly conjugated, macrocyclic structure (Figure 1). This structure yields large extinction coefficients especially in the blue region of the absorbance spectrum. The prominent electronic transitions of porphyrins and their metal complexes are the π→π* transitions associated with the macrocycle. Several studies have shown that cyclic compounds bind cofacially to this macrocycle [5] even when the compound bears a nitrogen [6] or the porphyrin bears a metal [7].

The semi-selective nature of porphyrin-based detection can impede the applicability of detection protocols to real-world scenarios. False-positive detection events can be reduced through careful selection of the porphyrin component so that detection relies on a shift (a peak/trough pair) in absorbance or fluorescence as opposed to a quench at a single wavelength. In order to further eliminate non-specific responses by the porphyrins, they can be used in conjunction with a scaffold that offers increased selectivity and protection of the indicators from irrelevant changes in the sensing environment. Porous silicate and organosilicate materials can be used to provide this protection. There are several reports of the use of mesoporous silicates as supports for porphyrin probes [8–18]. The International Union of Pure and Applied Chemistry (IUPAC) defines the prefix meso- as referring to a region 2 to 50 nm; macro- is a region >50 nm; and micro- is a region <2 nm. Mesopores limit the analytes that are admitted to the interior of the material and pore size can be controlled to provide the possibility of molecular sieving. Mesoporosity can also be used to provide a high surface area (potentially exceeding 1,000 m^2^/g) and pore volumes greater than 1 cm^3^/g. These materials characteristics offer the ability to immobilize a high concentration of indicators in a small area, thereby increasing the overall binding affinity of the construct. Organosilicate materials have been used to adsorb targets in aqueous solution as well as target vapors [19–21].

**Figure 2.**
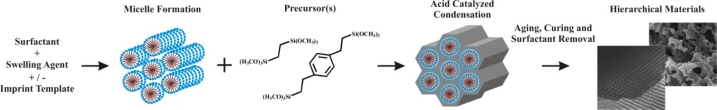
Synthesis of hierarchical macro/mesoporous organosilicate scaffolds.

The study presented here employed materials synthesized using a surfactant template approach for engineering porosity and organization on the meso-scale (Figure 2) [22–24]. In addition, polymerization (condensation)-induced phase separation has been used to produce macroporous frameworks which contain the ordered mesoporous structures [25–32]. The macroporous networks are intended to provide enhanced diffusion of targets within the materials and full access to the available surface area. The precursors used for materials synthesis consist of an organic moiety between two trialkoxysilane groups. The result is alternating siloxane and organic moieties in the pore walls of the materials that provide properties associated with both organic and inorganic materials [33,34]. The siloxane groups provide structural rigidity and hydrophilic character as well as the rugged character of a silicate material. The organic bridging groups provide binding characteristics normally associated with organic polymers. Through control of parameters during synthesis (precursors, surfactants, acids, *etc.*), both the structural and chemical properties of the materials can be tuned for a given application.

Adsorption of specific targets depends on the interaction of analytes with the surfaces of the porous materials. In these silicate materials, a type of molecular imprinting can be employed to provide areas on the surface of the materials which offer more favorable binding interactions. The process involves the introduction of a surfactant with a head-group structure similar to that of the target [18,35,36]. This target-like surfactant is incorporated as a fraction of the total surfactant in micelles around which the precursor materials are condensed. Upon extraction of the surfactant, sites that offer target complementary interactions remain in locations where the target-like surfactant was present. This approach has been shown to yield an increase in adsorption capacity and to increase the selectivity for a given target [35].

We have previously reported our efforts at combining the materials characteristics provided by organosilicate scaffolds with the optical and catalytic properties of porphyrins [18,36]. These efforts focused on nitroenergetic targets and achieved marginal success. In order to enhance the performance of the porphyrin-embedded materials (PEMs), the porous materials and the porphyrins used as well as the method of porphyrin incorporation into the scaffolds needed to be optimized. We recently reported on our efforts to optimize the structural characteristics of the materials [35]. Here, we describe the material characteristics developed for optimal paraoxon sorption, selection of porphyrin candidates, and the methods necessary for incorporation of functional porphyrin indicators into the materials. We also present results related to application of the constructs to detection of paraoxon.

## 2. Results and Discussion

The targets of interest to this study are organophosphates, organophosphonates, and those compounds of related structure such as the nerve agents sarin and VX. For the worked presented here, paraoxon has been used as a model compound. The design of a PEM for detection begins with the selection of organic bridging groups that will provide affinity for the target. Synthesis conditions must be established, surfactants and imprint templates selected, and surface modification addressed in order to complete the organosilicate scaffold. The porphyrin component must also be selected and a suitable method for incorporation of the fluorophore into the scaffold determined. The porphyrin should provide binding affinity for the target as well as strong changes in spectrophotometric characteristics upon target interaction. In general, optimization of scaffold and porphyrin components is completed in parallel. Once the necessary components have been developed, methods for porphyrin incorporation are evaluated and final modifications are made to optimize the complete construct.

### 2.1. Porphyrin Indicator

**Figure 3.**
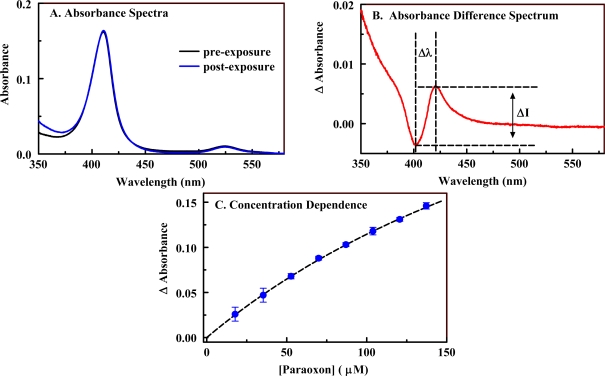
Changes in the porphyrin absorbance characteristics upon interaction with paraoxon. Panel A, absorbance spectra of Ni(II) C_1_S_3_TPP (20 μM) in the absence and presence of paraoxon (100 ppm) in 10 mM PBS. Panel B, difference spectrum calculated as post-exposure minus pre-exposure absorbance. In this spectrum, the distance between the peak position and the trough position is Δλ while the difference between the peak height and the trough depth is ΔI. Panel C, concentration dependence of the interaction between paraoxon and Cu(II) C_4_TPP (4 μM).

A number of metalloporphyrin variants were considered for their potential as indicators of the presence of paraoxon. Four porphyrin structures (Figure 1) were combined with 21 metals to provide a total of 84 candidates. A rapid screening process was applied to the 84 candidate porphyrins in which the changes in the absorbance spectrum for a single porphyrin and target concentration were evaluated. The interaction of a target with a porphyrin in solution results in a change in the π-bond conformation of the porphyrin macrocycle and yields spectrophotometric characteristics that are different from those of the porphyrin free in solution. In an absorbance spectrum, these changes can include increased or decreased absorbance at one or many wavelengths. In order to analyze these changes, it is useful to employ an absorbance difference spectrum calculated as the point-by-point subtraction of the pre-exposure spectrum from the post-exposure spectrum. Examples of pre- and post-exposure spectra as well as a difference spectrum are provided in Figure 3. Here, Ni(II) C_1_S_3_TPP (20 μM) was exposed to paraoxon (100 ppm). In the difference spectrum, the distance between the peak and the trough and the change in intensity (peak intensity minus the trough intensity) provide an indication of the binding affinity between the target and the porphyrin as well as the potential for application of the porphyrin as an indicator (sensitivity) [7]. Table 1 provides some examples of selected and rejected candidates. Selected candidates were those found to yield the largest Δλ values, ΔI values, or both. Ni(II) C_1_S_3_TPP demonstrated the most significant change in wavelength while Cu(II) C_4_TPP showed the most significant change in intensity.

Candidates selected using the rapid screening method described above were further screened through the generation of a binding isotherm using a fixed porphyrin concentration and a range of target concentrations (Figure 3). Here, changes in intensity for a given wavelength are plotted as a function of the target concentration. This data can be used to determine binding constants (K_11_) and changes in the extinction coefficients (Δɛ _11_) upon binding (Table 1) [37]. On the basis of these results, Ni(II) C_1_S_3_TPP, Ni(II) C_4_TPP, Ni(II) C_1_TPP, Fe(II) C_4_TPP, and Cu(II) C_4_TPP were selected for incorporation into the organosilicate scaffolds. The metal free versions of these porphyrins were also evaluated to further validate the selection process.

### 2.2. The Organosilicate Scaffold

**Figure 4.**
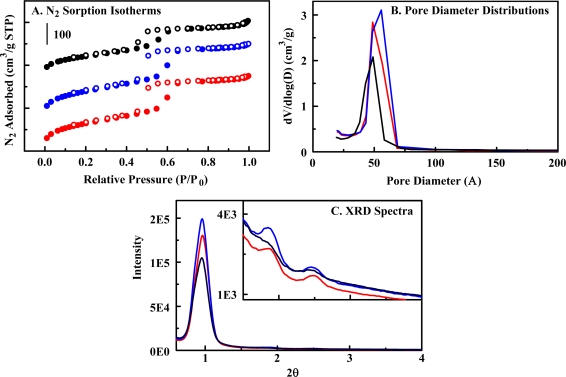
Structural characterization (Material **1**, red; Material **2**, blue; Material **5**, black). Panel A, nitrogen sorption isotherms offset by 0, 150, and 350 cm^3^/g, respectively. Panel B, pore size distributions. Panel C, XRD spectra (inset shows expansion of the 1.5 to 4 range).

**Figure 5.**
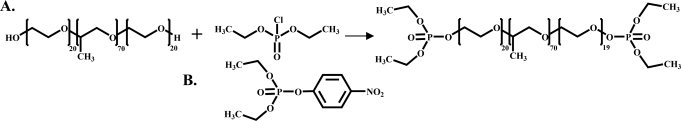
Esterification of the surfactant (**A**) to provide an analog of the paraoxon structure (**B**).

Based on previous work [38], diethylbenzene (DEB) bridging groups were selected to provide binding affinity and capacity for paraoxon. Two materials were synthesized in order to compare imprinted and non-imprinted material variants (Figure 4). Both materials demonstrated Type IV nitrogen sorption isotherms and possessed narrow pore size distributions. XRD spectra displayed a peak in the low angle region near 0.96° 2θ which could be attributed to the (100) reflection common for mesoporous materials with hexagonal order. Higher order reflections of extremely low intensity were observed. Transmission electron microscopy of previously reported materials showed hexagonal arrays of parallel pore channels [25]. The first material (Material **1**) was a co-condensate of 50% DEB and 1,2-bis(trimethoxysilyl)ethane (BTE). The inclusion of ethane bridges in co-condensates can yield enhanced structural characteristics and improves the effectiveness of the imprinting process used in the second material (Table 2) [35]. The process of imprinting the organosilicate materials involves the use of a surfactant with a modified head group. Here, esterification of Pluronic P123 using diethyl chlorophosphate (Figure 5) produces an analog of the phosphoric acid portion of the paraoxon structure. The goal of this process is to generate sites possessing more favorable interaction characteristics on the pore walls. The modified surfactant was included as 12.6% of the total surfactant used during synthesis of Material **2**. Conditions for synthesis, including concentrations of surfactant, acid, and swelling agent, were selected based on previous work [25].

**Figure 6.**
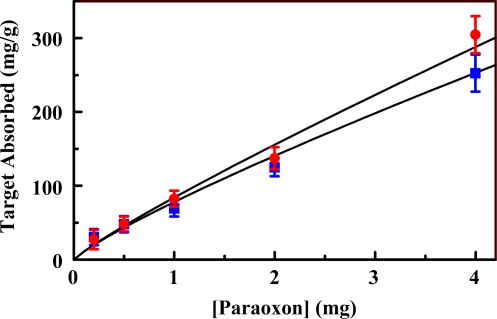
Paraoxon binding isotherms for imprinted (Material **2**, red) and non-imprinted (Material **1**, blue) organosilicate scaffolds.

Binding of paraoxon from aqueous solution by the organosilicate materials was evaluated to determine the binding capacity and target affinity provided by the scaffold. An HPLC difference method was applied in which the concentration of target in the sample is measured before and after exposure to the sorbent. The goal is for the scaffold to provide a locally high concentration of target in the vicinity of the porphyrin indicator. This should enhance the sensitivity of detection. We have previously shown that imprinting of the organosilicate materials enhances selective or semi-selective adsorption of analytes [18]. This effect should reduce the interaction of the porphyrin indicators with non-target compounds. Binding isotherms for Materials **1** and **2** are presented in Figure 6. Application of the imprinting process slightly increased the target bound by the material. Typically, the Langmuir-Freundlich model, a variant of the Langmuir model used to account for surface heterogeneity [39,40], is applied to these binding isotherms. In this case, the amount of target bound by the material is clearly in excess of that expected for single layer adsorption. Single layer adsorption is an assumption necessary to the typical model. Binding of 300 mg paraoxon per gram sorbent would be sufficient to fill nearly half of the available mesopore volume (based on N_2_ sorption). This binding capacity was far greater than expected. An effort is underway to understand this phenomenon, but those studies are beyond the scope of this manuscript.

### 2.3. The Fluorescent Construct

**Figure 7.**
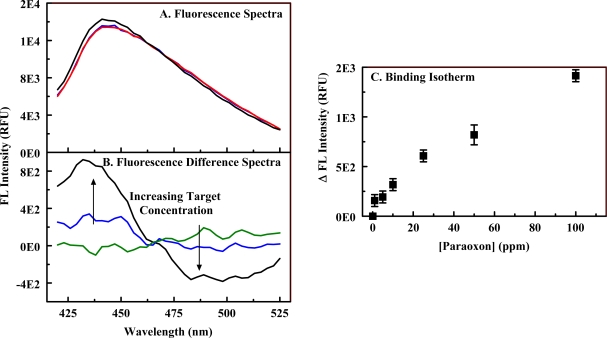
Interaction of paraoxon with Material **4**. Panel A, fluorescence excitation spectra for the Ni(II) C_1_S_3_TPP-embedded version of Material 4 in the presence/absence (red) of 5 ppm (blue) and 100 ppm (black) paraoxon. Panel B, difference fluorescence spectra for exposure of the material to 0.1 ppm (green), 10 ppm (blue), and 100 ppm (black). Panel C, binding isotherm for the interaction based on the peak/trough difference in intensity at 435 and 495 nm.

Porphyrins (C_1_S_3_TPP, C_4_TPP, and C_1_TPP) were incorporated into both imprinted and non-imprinted organosilicate scaffolds (Materials **3** and **4**) using EDC chemistry. Metalloporphyrin variants of the materials were generated by refluxing the porphyrin-embedded organosilicate in solutions of metals. These materials were synthesized with a low level of amine functionality in an attempt to preserve the binding characteristics of the organosilicate scaffold. In addition, excessively high loading of porphyrins onto a solid surface can result in co-facial stacking of the macrocycles. This type of stacking will significantly alter the spectrophotometric characteristics of the porphyrins as well as the response of the indicators to a target. The fluorescence response of the constructs to varying concentrations of paraoxon was evaluated in a manner similar to that described above (Section 2.1). Pre- and post-exposure fluorescence spectra were collected (Figure 7), and the difference spectra calculated. Ni(II) complexes of C_1_S_3_TPP, C_4_TPP, and C_1_TPP as well as Cu(II) and Fe(II) complexes of C_4_TPP were evaluated. Ni(II) C_1_S_3_TPP showed the highest sensitivity with the response in the imprinted material being enhanced over that of the non-imprinted material. A detection limit of 3 ppm was obtained for the Ni(II) C_1_S_3_TPP-embedded version of Material **4**. All porphyrin-embedded versions of Material **3** showed smaller changes in intensity than their Material **4** counter parts. The limit of detection (LOD) for paraoxon using Ni(II) C_4_TPP was 50 ppm in Material **3** versus 10 ppm in Material **4**. Fe(II) C_4_TPP showed dose dependence in the imprinted material but was not as sensitive as the Ni(II) C_1_S_3_TPP imprinted material. The Cu(II) C_4_TPP imprinted material showed dose dependence but with small changes in fluorescence. Limits of detection (LODs) and characteristic interaction wavelengths for several of these materials are provided in Table 3.

Given the high binding capacity of the scaffold for paraoxon, the high detection limit obtained with the PEMs (>3 ppm) tended to indicate the need for increased porphyrin loading in the construct. Because incorporation of high concentrations of 3-aminopropyltrimethoxysilane (APS) during synthesis causes disruption of the material structure, a grafting approach was used to increase the porphyrin loading level. Material **5** is the result of post-synthesis grafting of APS onto Material **2**. Using this approach, higher amine loading levels can be achieved, and the amine groups will be on the scaffold surfaces as opposed to also being distributed within the solid portion of the scaffold. UV/vis analysis of the porphyrin concentration remaining in solution following the immobilization reaction indicates loading of 3.0 weight percent porphyrin in Material **5**. The porphyrin loading level in Material **4** was approximately 0.5 weight percent.

**Figure 8.**
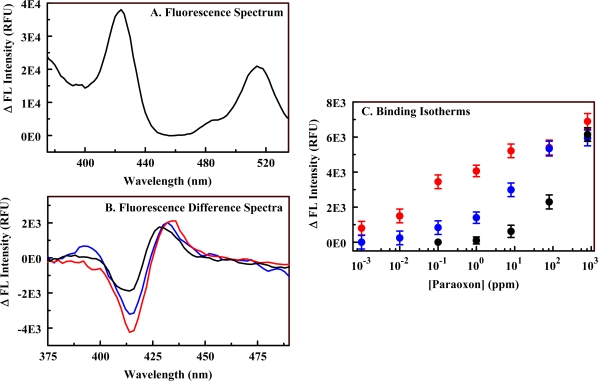
Interaction of paraoxon with Material **5**. Panel A, fluorescence excitation spectrum of Ni(II) C_1_S_3_TPP-embedded Material 5. Compare to Ni(II) C_1_S_3_TPP-embedded Material **4** (see Figure 7). Panel B, fluorescence difference spectra for exposure of Ni(II) C_1_S_3_TPP-embedded Material **5** to 0.1 (black), 8 (blue), and 800 (red) ppm paraoxon. Panel C, binding isotherm for Ni(II) C_1_S_3_TPP-embedded Material **5** (red) based on the peak/trough difference in intensity at 414 and 430 nm; binding isotherms for C_4_TPP (black)- and Ni(II) C_1_TPP (blue)-embedded versions of Material **5**.

The fluorescence responses to paraoxon exposure of porphyrin-embedded versions of Material **5** were evaluated. As shown in Figure 8, the change in intensity for a given exposure level is enhanced as a result of the increased porphyrin concentration (compare to Figure 7). This is expected as the change in absorbance/fluorescence is dependent on both analyte and target concentration [37]. The binding isotherms for the PEM materials (high and low amine loading) do not provide linear Benesi-Hilderbrand plots and, therefore, are not well represented by standard binding models typically applied to porphyrin-target interactions. Examples of target binding isotherms are provided in Figures 7 and 8. This deviation from standard models is not unexpected as both the affinity of the porphyrin and that of the scaffold contribute to the effective environment of the porphyrin indicators. Though a model for the binding interaction is not available, it is clear that increasing the porphyrin loading within the scaffolds provides enhanced target detection. The limit of detection for paraoxon using Ni(II) C_1_S_3_TPP in Material **5** (50 ppb) was significantly better than that for the porphyrin in Material **4** (3 ppm). Based on the experimental protocols used, experiments with Material **5** contained approximately 30 μg porphyrin while those with Material **4** contained only 5 μg for the same mass of PEM powder. Experiments in solution using Ni(II) C_1_S_3_TPP utilized 17 μg porphyrin and achieved a detection limit of 15 ppm. These differences in porphyrin concentration are not sufficient to fully account for the reduced detection limits noted with the PEM materials. This discrepancy implicates a contribution by the altered environment provided by the PEM.

Table 3 provides interaction wavelengths and limits of detection for the best performing PEMs as well as data sets that allow comparison of the performance of the different scaffolds. The characteristic wavelengths for the interaction of a given porphyrin with paraoxon were found to differ in several cases dependent upon the state of the porphyrin, ie., whether in solution or in a particular material. The interaction wavelengths for porphyrins embedded in Materials **3** and **4** were similar to each other but not to those observed in solution. The interaction wavelengths for porphyrins embedded in Material **5** differed from both the other materials and those of solution. For example, the characteristic interaction wavelengths for Ni(II) C_1_S_3_TPP in solution were at 402 and 408 nm; in Material **4**, they were 495 and 435 nm; in Material **5**, they were 414 and 430 nm. These differences are likely a result of variations in the environment of the porphyrins. When the excitation fluorescence spectra of the porphyrin in the various environments are compared, specific differences are noted. In solution the spectrum consists of a single, well defined peak centered at 412 nm (the Soret, Figure 3) and several less intense bands at longer wavelengths. When embedded in Materials **3** and **4**, the Soret is broadened and red-shifted to 444 nm (Figure 7). While a red-shift is consistent with the formation of porphyrin aggregates, the concentration of porphyrin in this material is well below the level that would be expected to cause stacking. These changes are more likely a result of the interaction of the porphyrin with the scaffold, perhaps the π-bonds of the diethylbenzene bridging groups. The porphyrin embedded in Material **5** has very different spectrophotometric characteristics. The Soret is centered around 424 nm, but there is an additional band at 515 nm. The intensity of the additional band is 60% of that for the band at 424 nm making it unlikely that this is a Q band for the porphyrin. Interaction between paraoxon and the porphyrin results in a change in absorbance related to the band at 424 nm, but not the band at 515 nm. The two bands are likely the result of porphyrin populations in two different environments. A portion of the embedded indicators see an environment that differs only slightly from the conditions experienced in solution (424 nm) while the other portion (515 nm) are either in an environment that is favorable to porphyrin stacking or to a strong interaction with the scaffold.

## 3. Experimental Section

Meso-tetra(4-carboxyphenyl) porphine (C_4_TPP); 5-mono(4-carboxyphenyl)-10, 15, 20-triphenyl porphine (C_1_TPP); meso-tri(4-sulfonatophenyl)mono(4-carboxyphenyl) porphine (C_1_S_3_TPP); and Deuteroporphyrin IX 2,4 bis-ethylene glycol (DIXEG) were obtained from Frontier Scientific, Logan, UT. Bis(trimethoxysilylethyl)benzene (DEB), 3-aminopropyltrimethoxysilane (APS) and 1,2-bis(trimethoxysilyl)ethane (BTE) were obtained from Gelest, Inc. (Tullytown, PA). Pluronic®P123 was generously donated by BASF. Diethyl chlorophosphate, dichloromethane (≥99.5%), magnesium turnings (98%), mesitylene (1,3,5-trimethylbenzene or TMB), and paraoxon were purchased from Sigma-Aldrich (St. Louis, MO). 1-Ethyl-3-[3-dimethylaminopropyl]carbodiimide (EDC) was purchased from Pierce Chemical Company (Rockford, IL). Chemicals were used as received. Water was deionized to 18.2 MΩ cm using a Millipore Milli Q UV-Plus water purification system.

The target analog used for imprinting was generated through esterification of Pluronic P123 (abbreviated P123 throughout) with diethyl chlorophosphate [41–43]. Briefly, P123 (4g; 0.7 mmol) and diethyl chlorophosphate (0.4 mL; 3 mmol) were dissolved in 60 mL of dichloromethane. Magnesium turnings were added and the mixture was refluxed for 3 h. The liquid was shaken with 60 mL 2% NaHCO_3_ in a separatory funnel. The organic phase was then extracted and evaporated under vacuum.

Our preparation method for the materials using P123 in acidic media has been described elsewhere [25]. Briefly, 1.9 g P123 (1.66 g P123 and 0.24 g imprint P123 for imprinted versions, Materials **2**, **4**, and **5**) and 0.55 g TMB were dissolved in 7.5 g 0.1 M HNO_3_ with stirring at 60 °C. The stirring solution was allowed to cool to room temperature and a silane mixture consisting of 0.00784 mol total bis silane (BTE + DEB) was added drop-wise. The reaction mixture was stirred until homogeneous and then transferred to a culture tube which was sealed tightly and heated at 60 °C over night (approximately 18 h). A white gel formed during this period. The tube was unsealed and heated at 60 °C for 2 d, and then 80 °C for 2 d. The product in the form of a white monolith was refluxed three times in ethanol for at least 12 h to extract P123, a process that rendered the monolith a powder. The material was collected by suction filtration, rinsed with ethanol and water, and dried at 100 °C.

Amine functional groups were directly incorporated in Materials **3** and **4** through inclusion of 0.05 g APS in the BTE-DEB silane mixture that was added drop-wise to the P123 solution. For Material **4**, the acid concentration was adjusted to 9.5 g 0.1 M HNO_3_. Material 5 was synthesized with 0.6 g TMB and 7.5 g 0.1 M HNO_3_ to obtain larger mesopore sizes which would better accommodate post-synthetic grafting of APS. Sorbent material (1.0 g) was dried at 110 °C and added to a solution of 1 mL APS in 100 mL toluene. The mixture was refluxed for 18 h. Grafted material was collected by vacuum filtration, washed with toluene and excess ethanol, and dried at 100 °C.

N_2_ sorption experiments were completed on a Micromeritics ASAP 2010 at 77 K. Samples were degassed to 1 μm Hg at 100 °C prior to analysis. Surface area was determined by use of the Brunauer-Emmett-Teller (BET) method; pore size was calculated by the Barrett-Joyner-Halenda (BJH) method from the adsorption branch of the isotherm; total pore volume was calculated by the single point method at relative pressure (P/P_0_) 0.97. Powder x-ray diffraction patterns were collected at room temperature using CuKα radiation from a Brüker MICROSTAR-H x-ray generator operated at 40 kV and 30 mA equipped with a 3 mRadian collimator, and a Brüker Platinum-135 CCD area detector. A custom fabricated beamstop was mounted on the detector to allow data collection to approximately 0.4° 2θ (approximately 210 Å) with a sample to detector distance of 30 cm. After unwarping the images the XRD^2^ plug-in was used to integrate the diffraction patterns from 0.5° to 8.4° 2θ.

Metalloporphyrin solutions were prepared by mixing porphyrin solutions with concentrated metal solutions to achieve a 1:1 molar ratio. The solutions were then heated at 90 °C until dry. Metalloporphyrins were resuspended to a final concentration of 2 mM in deionized water. Porphyrins were incorporated into the silicate materials through standard EDC chemistry. Silicate material (0.5 g) was added to a solution prepared using 9 mg EDC and 15 mg porphyrin in 100 mM MES buffer (40 mL). The solution was incubated with agitation overnight and rinsed with 5 mM sodium hydroxide, ethanol, and water. Metals were incorporated into the immobilized porphyrins using a reflux technique following incorporation into the scaffold. In all cases, a 1 mM solution of metal in deionized water was used for this process (300 mL, <500 mg sorbent).

Concentrations for analyte adsorption experiments are indicated in the text and figure captions. For HPLC analysis, solutions were filtered following incubation using 0.2 μm PTFE syringe filters to remove the adsorbent and the captured target. The solution was then analyzed and the amount adsorbed was calculated via difference method. Standard curves were generated for each set of experiments to verify sample concentrations and proper instrument function. The HPLC method was adapted from Gebreegzi, *et al.* [44] and employed a Shimadzu High Performance Liquid Chromatography (HPLC) system with dual-plunger parallel flow solvent delivery modules (LC-20AD) and an auto-sampler (SIL-20AC; 20 μL injection volume) coupled to a photodiode array detector (SPD-M20A; 277 nm detection). The stationary phase was a C18 stainless steel analytical column (Luna, 150 mm × 4.6 mm, 3 μm diameter; Phenomenex, Torrance, CA) with an isocratic 50:50 acetonitrile: 1% aqueous acetic acid mobile phase (1.5 mL/min).

Fluorescence excitation (655 nm emission) and emission spectra (collected at the wavelength with greatest extinction coefficient for the given porphyrin) of the porphyrin-embedded materials were collected in 96-well format with a Tecan XSafire monochromator-based microplate reader (1 nm resolution). Fluorescence spectra of the PEMs were collected for dry materials. Paraoxon exposure was accomplished by applying the target as a solution in acetonitrile and allowing the solvent to fully evaporate before collection of post-exposure fluorescence spectra. Controls were exposed to acetonitrile only (no target) to verify that this procedure did not impact the PEM spectra. Absorbance spectra of porphyrins in aqueous solution (10 mM phosphate buffered saline, PBS) were also collected using the plate reader (370 nm–770 nm at 2 nm resolution). Difference spectra are calculated as the point-by-point subtraction of the pre-exposure spectrum from the post-exposure spectrum. Concentration dependence data presented is based on the average of measurements conducted in triplicate. Fitting of the data was accomplished in PSI-Plot v 8.51 (Poly Software International, Inc.).

## 4. Conclusions

We have described the development of porphyrin-embedded organosilicate materials for application to the detection of paraoxon and compounds of related structure. These materials are synthesized using an imprinting technique that has been shown to enhance both the binding capacity of the scaffold and the selectivity of the sorbents for targets of specific structure [18]. Porphyrin candidates were selected based on a rapid screening protocol in which the changes in intensity and wavelength shifts at a given porphyrin and target concentration are compared. Down-selection of candidates based on these criteria is followed by more detailed characterization for final indicator selection. We have shown here that this approach provides the information necessary for selection of appropriate porphyrin candidates. Incorporation of selected porphyrin indicators into the materials at low loading levels (0.5 weight percent) was found to provide constructs with poor sensitivity to paraoxon resulting in high limits of detection. Increasing porphyrin loading levels resulted in enhanced construct sensitivity and significantly lower limits of detection. The complex fluorescence spectra of these constructs indicated that only a portion of the indicator incorporated into the scaffold was utilized in detection of paraoxon.

Our previous efforts were aimed at designing PEMs for the detection and photocatalytic conversion of nitroenergetics [18,36]. As in the current study, those efforts sought to combine the semi-selective binding characteristics and rugged nature of organosilicate materials with the spectrophotometric characteristics of porphyrins. Those initial efforts were only marginally successful. Here, we have shown that tuning of the materials including adjustment of porphyrin incorporation methods provide enhanced construct characteristics. The methods developed for this study provided significant enhancement, but the procedure needs to be further optimized. The materials used in this study have been synthesized with an open macrostructure which facilitates rapid diffusion of targets throughout the scaffold. The open structure also provides enhanced access to the available surface area, access that is often restricted in purely meso- or microporous materials. The materials used here were synthesized as powders which are readily applicable to column and packed-bed formats. In these formats the open structure reduces the back pressures typically encountered when using purely meso- or microporous materials.

## Figures and Tables

**Table 1.** Interaction of porphyrins with paraoxon in solution.

**Porphyrin**	**Δ λ**	**Δ I**	**K_11_ (1/mM)**	**Δε_11_ Peak (A/mM)**	**Δε_11_ Trough (A/mM)**
Selected Candidates					
Ni(II) C_1_S_3_TPP	19	0.010	0.66	57	4.3
Fe(II) C_4_TPP	12	0.010	0.97	160	19
Ni(II) C_4_TPP	11	0.006	3.1	43	11
Cu(II) C_4_TPP	14	0.019	3.1	120	38
Ni(II) C_1_TPP	N/A	0.432	4.1	N/A	220
Rejected Candidates					
Co(II) C_1_TPP	N/A	0.012	0.22	N/A	3.0
Fe(II) C_1_S_3_TPP	10	0.010	0.09	14	36
Pt(IV) C_4_TPP	8	0.002	0.06	5	63

**Table 2.** Material Characteristics.

**Material**	**Designation**	**BET Surface Area (m^2^/g)**	**BJH Pore Volume (cm^3^/g)**	**Average Pore Diameter (Å)**
50:50 DEB:BTE No Imprint	Material **1**	445	0.476	49
50:50 DEB:BTE Imprinted	Material **2**	478	0.527	56
100% DEB*	--	455	0.414	45
100% BTE*	--	800	>1	75
50:50 DEB:BTE No Imprint with amine functionality	Material **3**	648	0.587	49
50:50 DEB:BTE Imprinted with amine functionality	Material **4**	405	0.443	48
50:50 DEB:BTE Imprinted with high amine functionality	Material **5**	343	0.374	49

* 100% DEB and 100% BTE material characteristics are provided for comparison purposes [25].

**Table 3.** Interaction of porphyrin embedded materials with paraoxon. Peak and trough positions are taken from difference excitation spectra. Limits of detection are based on the concentration required to yield a ΔI of three times the noise in the measurement.

**Scaffold**	**Porphyrin**	**Peak (nm)**	**Trough (nm)**	**LOD (ppm)**
Material **3**	Fe(II) C_4_TPP	430	410	90
	Ni(II) C_4_TPP	416	438	50
	Ni(II) C_1_TPP	N/A	N/A	N/A*
Material **4**	Ni(II) C_1_S_3_TPP	495	435	3
	Ni(II) C_1_TPP	420	412	50
	Ni(II) C_4_TPP	417	433	10
Material **5**	Ni(II) C_1_S_3_TPP	414	430	0.05
	Ni(II) C_1_TPP	424	414	8
	C_4_TPP	420	N/A	50

* No change in fluorescence was observed for the measured range of concentrations, 0.1 ppm to 100 ppm.
